# Synthesis, biological evaluation and molecular modelling of 2,4-disubstituted-5-(6-alkylpyridin-2-yl)-1*H*-imidazoles as ALK5 inhibitors

**DOI:** 10.1080/14756366.2020.1734799

**Published:** 2020-03-12

**Authors:** Myoung-Soon Park, Hyun-Ju Park, Young Jae An, Joon Hun Choi, Geunyoung Cha, Hwa Jeong Lee, So-Jung Park, Purushottam M. Dewang, Dae-Kee Kim

**Affiliations:** a Graduate School of Pharmaceutical Sciences, College of Pharmacy, Ewha Womans University, Seoul, South Korea; b School of Pharmacy, Sungkyunkwan University, Suwon, South Korea

**Keywords:** 2,4-Disubstituted-5-(6-alkylpyridin-2-yl)-1*H*-imidazoles, ALK5 inhibition, cancer immunotherapeutic agent, docking

## Abstract

A series of 2,4-disubstituted-5-(6-alkylpyridin-2-yl)-1*H*-imidazoles, **7a–c**, **11a–h**, and **16a–h** has been synthesised and evaluated for their ALK5 inhibitory activity in an enzyme assay and in a cell-based luciferase reporter assay. Incorporation of a quinoxalin-6-yl moiety and a methylene linker at the 4- and 2-position of the imidazole ring, respectively, and a *m-*CONH_2_ substituent in the phenyl ring generated a highly potent and selective ALK5 inhibitor **11e**. Docking model of ALK5 in complex with **11e** showed that it fitted well in the ATP-binding pocket with favourable interactions.

## 1. Introduction

Transforming growth factor-β (TGF-β) is one of the most potent immunosuppressive cytokines in the tumour microenvironment^1^. Elevated serum levels of TGF-β commonly observed in patients with advanced colorectal cancer^2^, breast cancer^3^^,^^4^, bladder carcinoma^5^^,^^6^, prostate cancer^7^^,^^8^, malignant melanoma^9^, pancreatic ductal adenocarcinoma^10^, and hepatocellular carcinoma^11^ have been strongly associated with tumour progression and poor clinical outcome. Overexpression of TGF-β receptors has been implicated in cancer^12^. In patients with advanced hepatocellular carcinoma treated with the first clinically available ALK5 inhibitor, galunisertib (**1**)^13^, an approximately two-fold longer overall survival was observed in patients having a TGF-β1 response compared to patients who did not have a TGF-β1 response^14^. Therefore, TGF-β signalling pathway is an attractive target for development of cancer immunotherapeutic agents.

**Figure 1.**
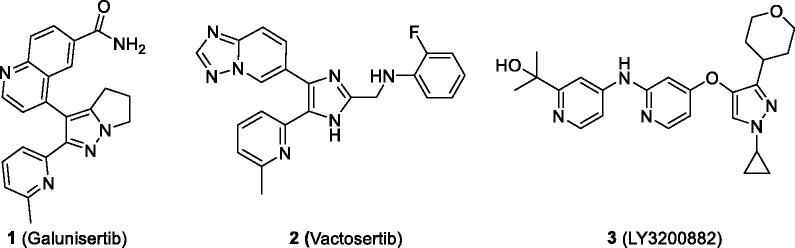
Small-molecule ATP-competitive ALK5 inhibitors in clinical trials.

TGF-β signals through two distinct serine/threonine kinase receptors, the type I (activin receptor-like kinase 5 (ALK5)) and type II receptors. Small-molecule ALK5 inhibitors specifically inhibit the Smad pathway by competing with ATP at the hydrophobic ATP binding pocket of ALK5 kinase domain, which is essential for the phosphorylation of its substrates, Smad2/Smad3 proteins. Galunisertib progressed to phase 2/3 clinical trials against pancreatic carcinoma, glioblastoma, hepatocellular carcinoma, and myelodysplastic syndrome^13^, however, Eli Lilly discontinued further clinical development of galunisertib in 2017. Recently, we developed an ALK5 inhibitor, vactosertib (**2**)^15^, and it has progressed to phase 1b/2a clinical trials either alone or in combination with pembrolizumab, durvalumab, or pomalidomide against myelodysplastic syndrome, non-small cell lung cancer, gastric cancer, colon cancer, multiple myeloma, etc.^16^. Another ALK5 inhibitor, LY3200882 (**3**) has entered a phase 1 clinical trial^16^ (Figure 1). Certain imidazo[2,1-*b*][1,3,4]thiadiazoles and 2,3,4-substituted 5,5-dimethyl-5,6-dihydro-4*H*-pyrrolo[1,2-*b*]pyrazoles have been reported to possess ALK5 inhibitory activity^17^^,^^18^.

Vactosertib exhibited subnanomolar ALK5 inhibitory activity in a kinase assay and in a cell-based luciferase reporter assay^15^, high selectivity against a panel of 320 protein kinases including p38α^15^, moderate oral bioavailability in rats^15^, and high efficacy in animal models of cancer^19–21^ and fibrosis^22–26^. In this report, we examined whether structural modification of vactosertib could increase its subnanomolar ALK5 inhibitory activity, thus further increasing its selectivity. For this purpose, we replaced a [1,2,4]triazolo[1,5-*a*]pyridin-6-yl moiety of vactosertib with either a benzo[1,3]dioxol-5-yl or a quinoxalin-6-yl moiety and inserted either a methylene, an ethylene, or a propylene linker instead of a methyleneamino linker to optimise the distance between a central imidazole ring and a phenyl ring.

## 2. Materials and methods

### 2.1. Chemistry

^1^H NMR spectra were recorded on a Varian Unity-Inova 400 MHz instrument. The chemical shifts are reported in parts per million (ppm). For ^1^H NMR spectra, CDCl_3_ was used as solvent, and it served as the internal standard at *δ* 7.26. Infra-red spectra were recorded on a FT-infra-red spectrometer (Bio-Rad). Electrospray ionisation mass spectra (ESIMS) were obtained on a Q-Tof2 mass spectrometer (Micromass). Elemental analyses (C, H, and N) were used to determine the purity of all tested compounds, and the results were within ±0.4% of the calculated values (Carlo Erba 1106 elemental analyzer). Analytical thin-layer chromatography (TLC) was performed on Merck silica gel 60 F-254 glass plates. Medium-pressure liquid chromatography (MPLC) was performed using Merck silica gel 60 (230–400 mesh) with a YFLC-540 ceramic pump (Yamagen).

#### 2.1.1. General procedure for the preparation of the 4-(benzo[1,3]dioxol-5-yl)-5–(6-methylpyridin-2-yl)-1*H*-imidazoles 6a–c

To a stirred solution of 1-(benzo[1,3]dioxol-5-yl)−2-(6-methylpyridin-2-yl)ethane-1,2-dione (**4**) (0.19 mmol) in AcOH (3 mL) were added NH_4_OAc (1.11 mmol) and 4-(2-oxoethyl)benzonitrile (**5a**), 4-(3-oxopropyl)benzonitrile (**5b**), or 4-(4-oxobutyl)benzonitrile (**5c**) (0.19 mmol), and the mixture was heated at 120 °C for 3 h. The pH of the cooled reaction mixture was adjusted to pH∼8 at 0 °C with 28% NH_4_OH solution in water, and the reaction mixture was extracted with CH_2_Cl_2_ (10 mL). The CH_2_Cl_2_ solution was washed with water (5 mL) and brine (5 mL), dried over anhydrous Na_2_SO_4_, filtered, and evaporated to dryness under reduced pressure. The residue was purified by MPLC on silica gel with CH_2_Cl_2_/MeOH as eluent to afford the **6a–c** as a solid.

##### 2.1.1.1. 4-((4-(Benzo[d][1,3]dioxol-5-yl)-5-(6-methylpyridin-2-yl)-1*H*-imidazol-2-yl)methyl)benzonitrile (6a)

Yield 30%; mp 176 − 179 °C; ^1^H NMR (CDCl_3_) δ 11.40 (br s, 1 H, NH), 7.45 (d, 2 H, 2 phenyl), 7.44 (overlapped, 1 H, pyridyl), 7.33 (d, 1 H, pyridyl), 7.25 (d, 2 H, 2 phenyl), 7.10 (d, 1 H, pyridyl), 7.07 (s, 1 H, piperonyl), 6.93 (d, 1 H, piperonyl), 6.84 (d, 1 H, piperonyl), 6.00 (s, 2 H, OCH_2_O), 4.09 (s, 2 H, CH_2_), 2.34 (s, 3 H, CH_3_); IR (CHCl_3_) 2229, 1574 cm^−1^; MS (EIS) *m/z* 395.13 (MH^+^).

##### 2.1.1.2. 4-(2-(4-(Benzo[d][1,3]dioxol-5-yl)-5-(6-methylpyridin-2-yl)-1*H*-imidazol-2-yl)ethyl)benzonitrile (6b)

Yield 49%; mp 94 − 96 °C; ^1^H NMR (CDCl_3_) δ 10.53 (br s, 1 H, NH), 7.56 (d, 2 H, 2 phenyl), 7.43 (dd, 1 H, pyridyl), 7.31 (d, overlapped, 1 H, pyridyl) 7.29 (d, 2 H, 2 phenyl), 7.07 (d, 1 H, pyridyl), 7.04 (d, 1 H, piperonyl), 6.94 (d, 1 H, piperonyl), 6.84 (d, 1 H, piperonyl), 6.00 (s, 2 H, OCH_2_O), 3.13 (m, 2 H, CH_2_), 3.05 (m, 2 H, CH_2_), 2.48 (s, 3 H, CH_3_); IR (CHCl_3_) 3375, 2229, 1573 cm^−1^; MS (EIS) *m/z* 409.13 (MH^+^).

##### 2.1.1.3. 4-(3-(4-(Benzo[d][1,3]dioxol-5-yl)-5-(6-methylpyridin-2-yl)-1*H*-imidazol-2-yl)propyl)benzonitrile (6c)

Yield 36%; mp 60 − 62 °C; ^1^H NMR (CDCl_3_) δ 10.63 (br s, 1 H, NH), 7.51 (d, 2 H, 2 phenyl), 7.42 (dd, 1 H, pyridyl), 7.30 (d, 1 H, pyridyl), 7.24 (d, 2 H, 2 phenyl), 7.10 − 7.04 (m, 2 H, 1 pyridyl and 1 piperonyl), 6.93 (d, 1 H, piperonyl), 6.82 (d, 1 H, piperonyl), 5.98 (s, 2 H, OCH_2_O), 2.74 (m, 4 H, 2 CH_2_), 2.47 (s, 3 H, CH_3_), 2.07 (m, 2 H, CH_2_); IR (CHCl_3_) 2228, 1573 cm^−1^; MS (EIS) *m/z* 423.14 (MH^+^).

#### 2.1.2. General procedure for the preparation of the 4-(benzo[1,3]dioxol-5-yl)-5-(6-methylpyridin-2-yl)-1*H*-imidazoles 7a–c

A stirred solution of **6a–c** (0.17 mmol), 6 N NaOH (0.04 mmol), and 28% H_2_O_2_ (0.59 mmol) in 95% EtOH (4 mL) was heated at 55 °C for 3 h. The reaction mixture was cooled to 0 °C and neutralised with 1 N HCl to pH∼8. The mixture was extracted with CH_2_Cl_2_ (30 mL), and the organic solution was washed with water (15 mL) and brine (15 mL), dried over anhydrous Na_2_SO_4_, filtered, and evaporated to dryness under reduced pressure. The residue was purified by MPLC on silica gel with CHCl_3_/MeOH or CH_2_Cl_2_/MeOH as eluent to afford the titled compounds **7a–c** as a solid.

##### 2.1.2.1. 4-((4-(Benzo[d][1,3]dioxol-5-yl)-5-(6-methylpyridin-2-yl)-1*H*-imidazol-2-yl)methyl)benzamide (7a)

Yield 55%; mp 248 − 250 °C; ^1^H NMR (CDCl_3_) δ 11.70 (br s, 1 H, NH), 7.45 (d, 2 H, 2 phenyl), 7.39 (dd, 1 H, pyridyl), 7.27 (d, 1 H, pyridyl), 7.10 (d, 2 H, 2 phenyl), 7.06 (overlapped, 1 H, pyridyl), 7.05 (s, 1 H, piperonyl), 6.87 (d, 1 H, piperonyl), 6.80 (d, 1 H, piperonyl), 6.39 (br s, 1 H, CONH), 5.95 (s, 2 H, OCH_2_O), 5.78 (br s, 1 H, CONH), 4.02 (s, 2 H, CH_2_), 2.27 (s, 3 H, CH_3_); IR (CHCl_3_) 3191, 1661 cm^−1^; MS (EIS) *m/z* 413.11 (MH^+^). Anal. Calcd for C_24_H_20_N_4_O_3_: C, 69.89; H, 4.89; N, 13.58. Found: C, 69.78; H, 4.95; N, 13.48.

##### 2.1.2.2. 4-(2-(4-(Benzo[d][1,3]dioxol-5-yl)-5-(6-methylpyridin-2-yl)-1*H*-imidazol-2-yl)ethyl)benzamide (7b)

Yield 49%; mp 189−192 °C; ^1^H NMR (CDCl_3_) δ 10.85 (br s, 1 H, NH) 7.66 (d, 2 H, 2 phenyl), 7.42 (t, 1 H, pyridyl), 7.30 (d, 1 H, pyridyl), 7.19 (d, 2 H, 2 phenyl), 7.07 (d, overlapped, 1 H, pyridyl), 7.05 (s, 1 H, piperonyl), 6.93 (d, 1 H, piperonyl), 6.82 (d, 1 H, piperonyl), 6.37 (br s, 1 H, CONH), 5.99 (s, 2 H, OCH_2_O), 5.69 (br s, 1 H, CONH), 3.04 (m, 2 H, CH_2_), 3.01 (m, 2 H, CH_2_), 2.45 (s, 3 H, CH_3_); IR (CHCl_3_) 3441, 1653 cm^−1^; MS (EIS) *m/z* 427.10 (MH^+^). Anal. Calcd for C_25_H_22_N_4_O_3_: C, 70.41; H, 5.20; N, 13.14. Found: C, 70.23; H, 5.28; N, 12.98.

##### 2.1.2.3. 4-(3-(4-(Benzo[d][1,3]dioxol-5-yl)-5-(6-methylpyridin-2-yl)-1*H*-imidazol-2-yl)propyl)benzamide (7c)

Yield 52%; mp 178−180 °C; ^1^H NMR (CDCl_3_) δ 10.60 (br s, 1 H, NH) 7.68 (d, 2 H, 2 phenyl), 7.41 (dd, 1 H, pyridyl), 7.30 (d, 1 H, pyridyl), 7.20 (d, 2 H, 2 phenyl), 7.10−7.05 (m, 2 H, 1 pyridyl and 1 piperonyl), 6.92 (d, 1 H, piperonyl), 6.82 (d, 1 H, piperonyl), 6.25 (br s, 1 H, CONH), 5.98 (s, 2 H, OCH_2_O), 5.75 (br s, 1 H, CONH), 2.73 (m, 4 H, 2 CH_2_), 2.49 (s, 3 H, CH_3_), 2.07 (m, 2 H, CH_2_); IR (CHCl_3_) 3183, 1660 cm^−1^; MS (EIS) *m/z* 441.12 (MH^+^). Anal. Calcd for C_26_H_24_N_4_O_3_: C, 70.89; H, 5.49; N, 12.72. Found: 70.63; H, 5.52; N, 12.66.

#### 2.1.3. General procedure for the preparation of the 5-(6-alkylpyridin-2-yl)-1-hydroxy-4-(quinoxalin-6-yl)-1*H*-imidazoles 10a–h

To a stirred solution of **9a–d** (0.23 mmol) in *t*-BuOMe (2.5 mL) were added either **5a** or 3-(2-oxoethyl)benzonitrile (**5d**) (0.69 mmol) and NH_4_OAc (1.15 mmol) dissolved in MeOH (1.2 mL), and the mixture was stirred at room temperature overnight under argon atmosphere. The pH of the reaction mixture was adjusted to pH∼8 at 0 °C with saturated NaHCO_3_ solution. The reaction mixture was partitioned between CH_2_Cl_2_ (40 mL) and water (40 mL). The aqueous layer was extracted with CH_2_Cl_2_ (15 mL × 3). The combined organic solution was dried over anhydrous Na_2_SO_4_, filtered, and evaporated to dryness under reduced pressure. The residue was purified by MPLC on silica gel with CH_2_Cl_2_/MeOH as eluent to afford the titled compounds **10a–h** as a solid.

##### 2.1.3.1. 4-((1-Hydroxy-5-(6-methylpyridin-2-yl)-4-(quinoxalin-6-yl)-1*H*-imidazol-2-yl)methyl)benzonitrile (10a)

Yield 40%; mp 200–203 °C; ^1^H NMR (CDCl_3_) δ 8.86 (m, 2 H, 2 quinoxalinyl), 8.35 (d, 1 H, quinoxalinyl), 8.17 (d, 1 H, quinoxalinyl), 8.06 (dd, 1 H, quinoxalinyl), 7.62 (m, 2 H, 2 phenyl), 7.56 (m, 2 H, 2 phenyl), 7.52 (t, 1 H, pyridyl), 7.36 (d, 1 H, pyridyl), 7.06 (d, 1 H, pyridyl), 4.31 (s, 2 H, CH_2_), 2.61 (s, 3 H, CH_3_); IR (CHCl_3_) 3075, 2228, 1600, 1572 cm^−1^; MS (EIS) *m/z* 419.23 (MH^+^).

##### 2.1.3.2. 4-((5-(6-Ethylpyridin-2-yl)-1-hydroxy-4-(quinoxalin-6-yl)-1*H*-imidazol-2-yl)methyl)benzonitrile (10b)

Yield 37%; mp 84–85 °C; ^1^H NMR (CDCl_3_) δ 8.86 (m, 2 H, 2 quinoxalinyl), 8.36 (d, 1 H, quinoxalinyl), 8.17 (d, 1 H, quinoxalinyl), 8.07 (dd, 1 H, quinoxalinyl), 7.62 (m, 2 H, 2 phenyl), 7.57 (m, 2 H, 2 phenyl), 7.54 (t, 1 H, pyridyl), 7.37 (d, 1 H, pyridyl), 7.08 (d, 1 H, pyridyl), 4.32 (s, 2 H, CH_2_), 2.90 (q, 2 H, CH_2_), 1.38 (t, 3 H, CH_3_); IR (CHCl_3_) 2229, 1599, 1572 cm^−1^; MS (EIS) *m/z* 433.18 (MH^+^).

##### 2.1.3.3. 4-((1-Hydroxy-5-(6-isopropylpyridin-2-yl)-4-(quinoxalin-6-yl)-1*H*-imidazol-2-yl)methyl)benzonitrile (10c)

Yield 46%; mp 178–179 °C; ^1^H NMR (CDCl_3_) δ 8.86 (m, 2 H, 2 quinoxalinyl), 8.36 (d, 1 H, quinoxalinyl), 8.17 (d, 1 H, quinoxalinyl), 8.07 (dd, 1 H, quinoxalinyl), 7.62 (m, 2 H, 2 phenyl), 7.57 (m, 2 H, 2 phenyl), 7.55 (t, 1 H, pyridyl), 7.37 (d, 1 H, pyridyl), 7.09 (d, 1 H, pyridyl), 4.32 (s, 2 H, CH_2_), 3.13 (heptet, 1 H, CH), 1.38 (s, 3 H, CH_3_), 1.37 (s, 3 H, CH_3_); IR (CHCl_3_) 2229, 1598, 1571 cm^−1^; MS (EIS) *m/z* 447.22 (MH^+^).

##### 2.1.3.4. 4-((5-(6-n-Butylpyridin-2-yl)-1-hydroxy-4-(quinoxalin-6-yl)-1*H*-imidazol-2-yl)methyl)benzonitrile (10d)

Yield 51%; mp 77–78 °C; ^1^H NMR (CDCl_3_) δ 8.86 (m, 2 H, 2 quinoxalinyl), 8.35 (d, 1 H, quinoxalinyl), 8.17 (d, 1 H, quinoxalinyl), 8.06 (dd, 1 H, quinoxalinyl), 7.62 (m, 2 H, 2 phenyl), 7.57 (m, 2 H, 2 phenyl), 7.53 (t, 1 H, pyridyl), 7.36 (dd, 1 H, pyridyl), 7.06 (d, 1 H, pyridyl), 4.31 (s, 2 H, CH_2_), 2.85 (t, 2 H, CH_2_), 1.76 (m, 2 H, CH_2_), 1.42 (m, 2 H CH_2_), 0.98 (t, 3 H, CH_3_); IR (CHCl_3_) 3402, 2228, 1608, 1572 cm^−1^; MS (EIS) *m/z* 461.20 (MH^+^).

##### 2.1.3.5. 3-((1-Hydroxy-5-(6-methylpyridin-2-yl)-4-(quinoxalin-6-yl)-1*H*-imidazol-2-yl)methyl)benzonitrile (10e)

Yield 77%; mp 210–212 °C; ^1^H NMR (CDCl_3_) δ 8.86 (m, 2 H, 2 quinoxalinyl), 8.36 (d, 1 H, quinoxalinyl), 8.18 (d, 1 H, quinoxalinyl), 8.08 (dd, 1 H, quinoxalinyl), 7.76 (s, 1 H, phenyl), 7.70 (d, 1 H, phenyl), 7.53 (m, 1 H, phenyl), 7.51 (d, 1 H, phenyl), 7.43 (t, 1 H, pyridyl), 7.37 (d, 1 H, pyridyl), 7.07 (d, 1 H, pyridyl), 4.29 (s, 2 H, CH_2_), 2.63 (s, 3 H, CH_3_); IR (CHCl_3_) 3052, 2231, 1575 cm^−1^; MS (EIS) *m/z* 419.20 (MH^+^).

##### 2.1.3.6. 3-((5-(6-Ethylpyridin-2-yl)-1-hydroxy-4-(quinoxalin-6-yl)-1*H*-imidazol-2-yl)methyl)benzonitrile (10f)

Yield 71%; mp 195–196 °C; ^1^H NMR (CDCl_3_) δ 8.85 (m, 2 H, 2 quinoxalinyl), 8.36 (d, 1 H, quinoxalinyl), 8.17 (d, 1 H, quinoxalinyl), 8.07 (dd, 1 H, quinoxalinyl), 7.75 (s, 1 H, phenyl), 7.70 (d, 1 H, phenyl), 7.54 (m, 2 H, 2 phenyl), 7.42 (t, 1 H, pyridyl), 7.38 (d, 1 H, pyridyl), 7.07 (d, 1 H, pyridyl), 4.29 (s, 2 H, CH_2_), 2.90 (q, 2 H, CH_2_), 1.38 (t, 3 H, CH_3_); IR (CHCl_3_) 2973, 2231, 1572 cm^−1^; MS (EIS) *m/z* 433.24 (MH^+^).

##### 2.1.3.7. 3-((1-Hydroxy-5-(6-isopropylpyridin-2-yl)-4-(quinoxalin-6-yl)-1*H*-imidazol-2-yl)methyl)benzonitrile (10g)

Yield 59%; mp 146–147 °C; ^1^H NMR (CDCl_3_) δ 8.86 (m, 2 H, 2 quinoxalinyl), 8.36 (d, 1 H, quinoxalinyl), 8.18 (d, 1 H, quinoxalinyl), 8.08 (dd, 1 H, quinoxalinyl), 7.76 (m, 1 H, phenyl), 7.71 (m, 1 H, phenyl), 7.54 (m, 2 H, 2 phenyl), 7.43 (t, 1 H, pyridyl), 7.38 (dd, 1 H, pyridyl), 7.10 (dd, 1 H, pyridyl), 4.30 (s, 2 H, CH_2_), 3.14 (heptet, 1 H, CH), 1.39 (s, 3 H, CH_3_), 1.37 (s, 3 H, CH_3_); IR (CHCl_3_) 2967, 2231, 1597, 1572 cm^−1^; MS (EIS) *m/z* 447.22 (MH^+^).

##### 2.1.3.8. 3-((5-(6-*n*-Butylpyridin-2-yl)-1-hydroxy-4-(quinoxalin-6-yl)-1*H*-imidazol-2-yl)methyl)benzonitrile (10h)

Yield 67%; mp 164–166 °C; ^1^H NMR (CDCl_3_) δ 8.85 (m, 2 H, 2 quinoxalinyl), 8.35 (d, 1 H, quinoxalinyl), 8.17 (d, 1 H, quinoxalinyl), 8.07 (dd, 1 H, quinoxalinyl), 7.75 (s, 1 H, phenyl), 7.70 (m, 1 H, phenyl), 7.53 (m, 2 H, 2 phenyl), 7.42 (t, 1 H, pyridyl), 7.37 (d, 1 H, pyridyl), 7.05 (d, 1 H, pyridyl), 4.29 (s, 2 H, CH_2_), 2.85 (t, 2 H, CH_2_), 1.76 (m, 2 H, CH_2_), 1.43 (m, 2 H, CH_2_), 0.98 (t, 3 H, CH_3_); IR (CHCl_3_) 2957, 2230, 1599, 1572 cm^−1^; MS (EIS) *m/z* 461.27 (MH^+^).

#### 2.1.4. General procedure for the preparation of the 5-(6-alkylpyridin-2-yl)-4-(quinoxalin-6-yl)-1*H*-imidazoles 11a–h

To a stirred solution of **10a–h** (0.63 mmol) in a mixture of EtOH (16 mL) and DMSO (4 mL) at room temperature were added 28% H_2_O_2_ (6.62 mmol) and 6 N NaOH (0.47 mmol). The mixture was warmed to 55 °C and stirred overnight, and to it, 1 N HCl solution was added to adjust to pH∼8 at 0 °C. The ethanol solvent was evaporated off under reduced pressure, and the residue was partitioned between CH_2_Cl_2_ (30 mL) and H_2_O (50 mL). The aqueous layer was saturated with NaCl and extracted with CH_2_Cl_2_ (30 mL × 3). The combined organic solution was washed with brine (30 mL), dried over anhydrous Na_2_SO_4_, filtered, and evaporated to dryness under reduced pressure. The residue was dissolved in anhydrous DMF (20 mL) and treated with triethyl phosphite (2.39 mmol). The mixture was heated at 110 °C for 3 days, cooled to room temperature, and evaporated to dryness under reduced pressure. The reaction mixture was partitioned between CH_2_Cl_2_ (30 mL) and water (50 mL), and the aqueous layer was extracted with CH_2_Cl_2_ (30 mL × 2). The combined organic solution was washed with saturated NaHCO_3_ solution (40 mL) and brine (50 mL), dried over anhydrous Na_2_SO_4_, filtered, and evaporated to dryness under reduced pressure. The residue was purified by MPLC on silica gel with CH_2_Cl_2_/MeOH as eluent to afford the titled compounds **11a–h** as a solid.

##### 2.1.4.1. 4-((5-(6-Methylpyridin-2-yl)-4-(quinoxalin-6-yl)-1*H*-imidazol-2-yl)methyl)benzamide (11a)

Yield 36%; mp 227–229 °C; ^1^H NMR (CDCl_3_) δ 12.01 (br s, 1 H, NH), 8.83 (m, 2 H, 2 quinoxalinyl), 8.38 (s, 1 H, quinoxalinyl), 8.15 (dd, 2 H, 2 quinoxalinyl), 7.55 (d, 2 H, 2 phenyl), 7.42 (dd, 1 H, pyridyl), 7.33 (d, 1 H, pyridyl), 7.21 (d, 2 H, 2 phenyl), 6.95 (d, 1 H, pyridyl), 6.62 (br s, 1 H, CONH), 5.83 (br s, 1 H, CONH), 4.13 (s, 2 H, CH_2_), 2.29 (s, 3 H, CH_3_); IR (CHCl_3_) 3185, 1665, 1616, 1572 cm^−1^; MS (EIS) *m/z* 421.14 (MH^+^). Anal. Calcd for C_25_H_20_N_6_O: C, 71.41; H, 4.79; N, 19.99. Found: C, 71.44; H, 4.65; N, 19.87.

##### 2.1.4.2. 4-((5-(6-Ethylpyridin-2-yl)-4-(quinoxalin-6-yl)-1*H*-imidazol-2-yl)methyl)benzamide (11 b)

Yield 44%; mp 218–219 °C; ^1^H NMR (CDCl_3_) δ 11.62 (br s, 1 H, NH), 8.83 (s, 2 H, 2 quinoxalinyl), 8.39 (s, 1 H, quinoxalinyl), 8.15 (dd, 2 H, quinoxalinyl), 7.59 (d, 2 H, 2 phenyl), 7.45 (t, 1 H, pyridyl), 7.33 (d, 1 H, pyridyl), 7.26 (d, 2 H, 2 phenyl), 6.99 (d, 1 H, pyridyl), 6.45 (br s, 1 H, CONH), 5.83 (br s, 1 H, CONH), 4.17 (s, 2 H, CH_2_), 2.65 (q, 2 H, CH_2_), 1.09 (t, 3 H, CH_3_); IR (CHCl_3_) 3179, 1663, 1615, 1571 cm^−1^; MS (EIS) *m/z* 435.19 (MH^+^). Anal. Calcd for C_26_H_22_N_6_O: C, 71.87; H, 5.10; N, 19.34. Found: C, 71.57; H, 5.28; N, 19.12.

##### 2.1.4.3. 4-((5-(6-Isopropylpyridin-2-yl)-4-(quinoxalin-6-yl)-1*H*-imidazol-2-yl)methyl)benzamide (11c)

Yield 35%; mp 151–152 °C; ^1^H NMR (CDCl_3_) δ 10.33 (br s, 1 H, NH), 8.84 (m, 2 H, 2 quinoxalinyl), 8.42 (s, 1 H, quinoxalinyl), 8.15 (s, 2 H, 2 quinoxalinyl), 7.76 (m, 2 H, 2 phenyl), 7.44 (m, 3 H, 2 phenyl and 1 pyridyl), 7.33 (d, 1 H pyridyl), 7.01 (d, 1 H, pyridyl), 6.10 (br s, 1 H, CONH), 5.60 (br s, 1 H, CONH), 4.28 (s, 2 H, CH_2_), 3.01 (heptet, 1 H, CH), 1.27 (s, 3 H, CH_3_), 1.25 (s, 3 H, CH_3_); IR (CHCl_3_) 3183, 1664, 1615, 1571 cm^−1^; MS (EIS) m/z 449.23 (MH^+^). Anal. Calcd for C_27_H_24_N_6_O: C, 72.30; H, 5.39; N, 18.74. Found: C, 72.25; H, 5.45; N, 18.61.

##### 2.1.4.4. 4-((5-(6-*n*-Butylpyridin-2-yl)-4-(quinoxalin-6-yl)-1*H*-imidazol-2-yl)methyl)benzamide (11d)

Yield 42%; mp 170–171 °C; ^1^H NMR (CDCl_3_) δ 11.47 (br s, 1 H, NH), 8.84 (s, 2 H, 2 quinoxalinyl), 8.40 (s, 1 H, quinoxalinyl), 8.16 (m, 2 H, 2 quinoxalinyl), 7.61 (d, 2 H, 2 phenyl), 7.43 (t, 1 H, pyridyl), 7.33 (d, 1 H, pyridyl), 7.28 (d, 2 H, 2 phenyl), 6.97 (d, 1 H, pyridyl), 6.35 (br s, 1 H, CONH), 5.68 (br s, 1 H, CONH), 4.19 (s, 2 H, CH_2_), 2.64 (t, 2 H, CH_2_), 1.45 (m, 2 H, CH_2_), 1.24 (m, 2 H, CH_2_), 0.81 (t, 3 H, CH_3_); IR (CHCl_3_) 3182, 1661, 1616, 1570 cm^−1^; MS (EIS) *m/z* 463.24 (MH^+^). Anal. Calcd for C_28_H_26_N_6_O: C, 72.71; H, 5.67; N, 18.17. Found: C, 72.83; H, 5.56; N, 18.02.

##### 2.1.4.5. 3-((5-(6-Methylpyridin-2-yl)-4-(quinoxalin-6-yl)-1*H*-imidazol-2-yl)methyl)benzamide (11e)

Yield 23%; mp 181 − 182 °C; ^1^H NMR (CDCl_3_) δ 8.79 (s, 2 H, 2 quinoxalinyl), 8.34 (s, 1 H, quinoxalinyl), 8.06 (s, 2 H, 2 quinoxalinyl), 7.71 (s, 1 H, phenyl), 7.55 (d, 1 H, phenyl), 7.41 (t, 1 H, pyridyl), 7.34 (d, 1 H, pyridyl), 7.29 (d, 1 H, phenyl), 7.19 (t, 1 H, phenyl), 6.96 (d, 1 H, pyridyl), 6.83 (br s, 1 H, CONH), 6.30 (br s, 1 H, CONH), 4.13 (s, 2 H, CH_2_), 2.36 (s, 3 H, CH_3_); IR (CHCl_3_) 3196, 1674, 1658 cm^−1^; MS (EIS) *m/z* 421.23 (MH^+^). Anal. Calcd for C_25_H_20_N_6_O: C, 71.41; H, 4.79; N, 19.99. Found: C, 71.26; H, 4.92; N, 19.85.

##### 2.1.4.6. 3-((5-(6-Ethylpyridin-2-yl)-4-(quinoxalin-6-yl)-1*H*-imidazol-2-yl)methyl)benzamide (11f)

Yield 26%; mp 125 − 126 °C; ^1^H NMR (CDCl_3_) δ 8.84 (m, 2 H, 2 quinoxalinyl), 8.40 (s, 1 H, quinoxalinyl), 8.13 (d, 2 H, 2 quinoxalinyl), 7.85 (s, 1 H, phenyl), 7.72 (m, 1 H, phenyl), 7.57 (m, 1 H, phenyl), 7.44 (m, 2 H, phenyl and pyridyl), 7.34 (br d, 1 H, pyridyl), 7.00 (dd, 1 H, pyridyl), 6.20 (br s, 1 H, CONH), 5.60 (br s, 1 H, CONH), 4.28 (s, 2 H, CH_2_), 2.80 (q, 2 H, CH_2_), 1.28 (t, 3 H, CH_3_); IR (CHCl_3_) 3407, 1670, 1657 cm^−1^; MS (EIS) *m/z* 435.22 (MH^+^). Anal. Calcd for C_26_H_22_N_6_O: C, 71.87; H, 5.10; N, 19.34. Found: C, 71.89; H, 5.15; N, 19.24.

##### 2.1.4.7. 3-((5-(6-Isopropylpyridin-2-yl)-4-(quinoxalin-6-yl)-1*H*-imidazol-2-yl)methyl)benzamide (11g)

Yield 29%; mp 114−115 °C; ^1^H NMR (CDCl_3_) δ 8.81 (m, 2 H, 2 quinoxalinyl), 8.37 (s, 1 H, quinoxalinyl), 8.11 (m, 2 H, 2 quinoxalinyl), 7.71 (s, 1 H, phenyl), 7.65 (m, 1 H, phenyl), 7.50 (d, 1 H, phenyl), 7.46 (t, 1 H, pyridyl), 7.36 (t, 1 H, phenyl), 7.35 (overlapped, 1 H, pyridyl), 7.02 (dd, 1 H, pyridyl), 6.37 (br s, 1 H, CONH), 5.70 (br s, 1 H, CONH), 4.22 (s, 2 H, CH_2_), 2.99 (heptet, 1 H, CH), 1.24 (s, 3 H, CH_3_), 1.22 (s, 3 H, CH_3_); IR (CHCl_3_) 3184, 1665, 1574 cm^−1^; MS (EIS) *m/z* 449.25 (MH^+^). Anal. Calcd for C_27_H_24_N_6_O: C, 72.30; H, 5.39; N, 18.74. Found: C, 72.03; H, 5.52; N, 18.67.

##### 2.1.4.8. 3-((5-(6-*n*-Butylpyridin-2-yl)-4-(quinoxalin-6-yl)-1*H*-imidazol-2-yl)methyl)benzamide (11h)

Yield 27%; mp 110−111 °C; ^1^H NMR (CDCl_3_) δ 8.83 (m, 2 H, 2 quinoxalinyl), 8.41 (s, 1 H, quinoxalinyl), 8.13 (s, 2 H, 2 quinoxalinyl), 7.81 (s, 1 H, phenyl), 7.70 (d, 1 H, phenyl), 7.55 (d, 1 H, phenyl), 7.42 (m, 2 H, phenyl and pyridyl), 7.32 (br d, 1 H, pyridyl), 6.98 (d, 1 H, pyridyl), 6.20 (br s, 1 H, CONH), 5.60 (br s, 1 H, CONH), 4.27 (s, 2 H, CH_2_), 2.77 (t, 2 H), 1.63 (m, 2 H, CH_2_), 1.38 (m, 2 H, CH_2_), 0.93 (t, 3 H, CH_3_); IR (CHCl_3_) 3183, 1666, 1576 cm^−1^; MS (EIS) *m/z* 463.25 (MH^+^). Anal. Calcd for C_28_H_26_N_6_O: C, 72.71; H, 5.67; N, 18.17. Found: C, 72.89; H, 5.51; N, 18.03.

#### 2.1.5. General procedure for the preparation of the 4-(3-oxopropyl)benzamide (14a) and 3-(3-oxopropyl)benzamide (14b)

To a stirred solution of 4-(2-(1,3-dioxolan-2-yl)ethyl)benzonitrile (**12a**) (1.50 g, 7.34 mmol) in MeOH (50 mL) at room temperature were added 28% H_2_O_2_ (25.70 mmol) and 6 N NaOH (7.34 mmol). The mixture was warmed to 55 °C and stirred for 2 h, and to it, 1 N HCl solution was added to adjust to pH∼8 at 0 °C. The MeOH was evaporated off under reduced pressure, and the residue was extracted with CH_2_Cl_2_ (30 mL × 3). The organic solution was washed with brine (30 mL), dried over anhydrous Na_2_SO_4_, filtered, and evaporated to dryness under reduced pressure. The residue was purified by MPLC on silica gel with MeOH/CH_2_Cl_2_ (1:19, then 1:9 (v/v)) as eluent to give 1.58 g (97%) of 4-(2-(1,3-dioxolan-2-yl)ethyl)benzamide (**13a**) as a solid. To a stirred solution of **13a** (0.50 g, 2.26 mmol) in THF (22 mL) was added 1 N HCl solution (20 mL) at room temperature. The mixture was heated under reflux for 1 h and cooled to room temperature. After saturation with NaCl, the reaction mixture was extracted with CHCl_3_ (20 mL × 5). The combined organic solution was dried over anhydrous Na_2_SO_4_, filtered, and evaporated under reduced pressure to give 0.40 g (98%) of 4-(3-oxopropyl)benzamide (**14a**) as a solid which was used to the next step without further purification.

The 3-(3-oxopropyl)benzamide (**14 b**) was prepared by the same procedure as for **14a**.

#### 2.1.6. General procedure for the preparation of the 5-(6-alkylpyridin-2-yl)-4-(quinoxalin-6-yl)-1*H*-imidazoles 16a–h

To a stirred solution of **15a–d** (3.79 mmol) in a mixture of *t*-BuOMe (35 mL) and MeOH (25 mL) were added either **14a** or **14b** (5.69 mmol) and NH_4_OAc (18.95 mmol), and the mixture was stirred at 30 °C overnight under argon atmosphere. The pH of the reaction mixture was adjusted to pH∼8 at 0 °C with saturated NaHCO_3_ solution. After removal of solvent, the reaction mixture was extracted with CH_2_Cl_2_ (25 mL × 3), and the organic solution was dried over anhydrous Na_2_SO_4_, filtered and evaporated to dryness under reduced pressure. The residue was purified by MPLC on silica gel with CH_2_Cl_2_/MeOH as eluent to afford the titled compounds **16a–h** as a solid.

##### 2.1.6.1. 4-(2-(5-(6-Methylpyridin-2-yl)-4-(quinoxalin-6-yl)-1*H*-imidazol-2-yl)ethyl)benzamide (16a)

Yield 66%; mp 145–147 °C; ^1^H NMR (CDCl_3_) δ 8.82 (m, 2 H, 2 quinoxalinyl), 8.36 (s, 1 H, quinoxalinyl), 8.09 (d, 2 H, 2 quinoxalinyl), 7.64 (d, 2 H, 2 phenyl), 7.43 (t, 1 H, pyridyl), 7.32 (d, 1 H, pyridyl), 7.17 (d, 2 H, 2 phenyl), 6.99 (d, 1 H, pyridyl), 6.58 (br s, 1 H, CONH), 6.09 (br s, 1 H, CONH), 3.06 (s, 4 H, 2 CH_2_), 2.45 (s, 3 H, CH_3_); IR (CHCl_3_) 3191, 1674, 1615, 1572 cm^−1^; MS (EIS) *m/z* 435.19 (MH^+^). Anal. Calcd for C_26_H_22_N_6_O: C, 71.87; H, 5.10; N, 19.34. Found: C, 71.65; H, 5.23; N, 19.30.

##### 2.1.6.2. 4-(2-(5-(6-Ethylpyridin-2-yl)-4-(quinoxalin-6-yl)-1*H*-imidazol-2-yl)ethyl)benzamide (16b)

Yield 63%; mp 123–125 °C; ^1^H NMR (CDCl_3_) δ 11.40 (br s, 1 H, NH), 8.82 (m, 2 H, 2 quinoxalinyl), 8.37 (s, 1 H, quinoxalinyl), 8.11 (m, 2 H, 2 quinoxalinyl), 7.64 (d, 2 H, 2 phenyl), 7.47 (t, 1 H, pyridyl), 7.35 (br d, 1 H, pyridyl), 7.19 (d, 2 H, 2 phenyl), 7.01 (d, 1 H, pyridyl), 6.50 (br s, 1 H, CONH), 5.90 (br s, 1 H, CONH), 3.08 (s, 4 H, 2 CH_2_), 2.74 (q, 2 H, CH_2_), 1.19 (t, 3 H, CH_3_); IR (CHCl_3_) 3418, 1666, 1570 cm^−1^; MS (EIS) *m/z* 449.20 (MH^+^). Anal. Calcd for C_27_H_24_N_6_O: C, 72.30; H, 5.39; N, 18.74. Found: C, 72.55; H, 5.26; N, 18.61.

##### 2.1.6.3. 4-(2-(5-(6-Isopropylpyridin-2-yl)-4-(quinoxalin-6-yl)-1*H*-imidazol-2-yl)ethyl)benzamide (16c)

Yield 71%; mp 113–115 °C; ^1^H NMR (CDCl_3_) δ 10.75 (br s, 1 H, NH), 8.83 (m, 2 H, 2 quinoxalinyl), 8.38 (s, 1 H, quinoxalinyl), 8.12 (m, 2 H, 2 quinoxalinyl), 7.70 (m, 2 H, 2 phenyl), 7.46 (t, 1 H, pyridyl), 7.34 (br s, 1 H, pyridyl), 7.27 (d, 2 H, 2 phenyl), 7.02 (d, 1 H, pyridyl), 6.33 (br s, 1 H, CONH), 5.85 (br s, 1 H, CONH), 3.14 (m, 4 H, 2 CH_2_), 3.00 (heptet, 1 H, CH), 1.26 (s, 3 H, CH_3_), 1.24 (s, 3 H, CH_3_); IR (CHCl_3_) 3446, 1652, 1626 cm^−1^; MS (EIS) *m/z* 463.21 (MH^+^). Anal. Calcd for C_28_H_26_N_6_O: C, 72.71; H, 5.67; N, 18.17. Found: C, 72.53; H, 5.82; N, 18.11.

##### 2.1.6.4. 4-(2-(5-(6-*n*-Butylpyridin-2-yl)-4-(quinoxalin-6-yl)-1*H*-imidazol-2-yl)ethyl)benzamide (16d)

Yield 57%; mp 100–102 °C; ^1^H NMR (CDCl_3_) δ 11.55 (br s, 1 H, NH), 8.83 (m, 2 H, 2 quinoxalinyl), 8.36 (s, 1 H, quinoxalinyl), 8.11 (m, 2 H, 2 quinoxalinyl), 7.62 (d, 2 H, 2 phenyl), 7.46 (t, 1 H, pyridyl), 7.36 (br d, 1 H, pyridyl), 7.18 (d, 2 H, 2 phenyl), 7.00 (dd, 1 H, pyridyl), 6.50 (br s, 1 H, CONH), 5.80 (br s, 1 H, CONH), 3.07 (s, 4 H, 2 CH_2_), 2.69 (t, 2 H, CH_2_), 1.54 (m, 2 H, CH_2_), 1.27 (m, 2 H, CH_2_), 0.82 (t, 3 H, CH_3_); IR (CHCl_3_) 3411, 1657, 1621, 1569 cm^−1^; MS (EIS) *m/z* 477.23 (MH^+^). Anal. Calcd for C_29_H_28_N_6_O: C, 73.09; H, 5.92; N, 17.63. Found: C, 72.98; H, 5.85; N, 17.71.

##### 2.1.6.5. 3-(2-(5-(6-Methylpyridin-2-yl)-4-(quinoxalin-6-yl)-1*H*-imidazol-2-yl)ethyl)benzamide (16e)

Yield 16%; mp 120−121 °C; ^1^H NMR (CDCl_3_) δ 8.82 (m, 2 H, 2 quinoxalinyl), 8.35 (s, 1 H, quinoxalinyl), 8.08 (s, 2 H, 2 quinoxalinyl), 7.62 (m, 2 H, 2 phenyl), 7.43 (t, 1 H, pyridyl), 7.32 (m, 2 H, phenyl and pyridyl), 7.29 (d, 1 H, phenyl), 6.99 (d, 1 H, pyridyl), 6.63 (br s, 1 H, CONH), 6.10 (br s, 1 H, CONH), 3.08 (m, 4 H, 2 CH_2_), 2.46 (s, 3 H, CH_3_); IR (CHCl_3_) 3192, 1658, 1581 cm^−1^; MS (EIS) *m/z* 435.20 (MH^+^). Anal. Calcd for C_26_H_22_N_6_O: C, 71.87; H, 5.10; N, 19.34. Found: C, 71.53; H, 5.35; N, 19.21.

##### 2.1.6.6. 3-(2-(5-(6-Ethylpyridin-2-yl)-4-(quinoxalin-6-yl)-1*H*-imidazol-2-yl)ethyl)benzamide (16f)

Yield 16%; mp 119−121 °C; ^1^H NMR (CDCl_3_) δ 8.80 (s, 2 H, 2 quinoxalinyl), 8.34 (t, 1 H, quinoxalinyl), 8.06 (s, 2 H, 2 quinoxalinyl), 7.59 (m, 2 H, 2 phenyl), 7.47 (t, 1 H, pyridyl), 7.33 (d, 1 H, pyridyl), 7.23 (t, 1 H, phenyl), 7.01 (dd, 1 H, pyridyl), 6.80 (br s, 1 H, CONH), 6.25 (br s, 1 H, CONH), 3.06 (m, 2 H, CH_2_), 3.01 (m, 2 H, CH_2_), 2.71 (q, 2 H, CH_2_), 1.16 (t, 3 H, CH_3_); IR (CHCl_3_) 3317, 1657, 1581 cm^−1^; MS (EIS) *m/z* 449.26 (MH^+^). Anal. Calcd for C_27_H_24_N_6_O: C, 72.30; H, 5.39; N, 18.74. Found: C, 72.44; H, 5.25; N, 18.58.

##### 2.1.6.7. 3-(2-(5-(6-Isopropylpyridin-2-yl)-4-(quinoxalin-6-yl)-1*H*-imidazol-2-yl)ethyl)benzamide (16g)

Yield 20%; mp 116−119 °C; ^1^H NMR (CDCl_3_) δ 8.82 (s, 2 H, 2 quinoxalinyl), 8.37 (s, 1 H, quinoxalinyl), 8.10 (m, 2 H, 2 quinoxalinyl), 7.69 (t, 1 H, phenyl), 7.62 (m, 1 H, phenyl), 7.46 (t, 1 H, pyridyl), 7.34 (m, 2 H, phenyl and pyridyl), 7.31 (t, 1 H, phenyl), 7.02 (dd, 1 H, pyridyl), 6.50 (br s, 1 H, CONH), 6.00 (br s, 1 H, CONH), 3.13 (s, 4 H, 2 CH_2_), 3.00 (heptet, 1 H, CH), 1.25 (s, 3 H, CH_3_), 1.24 (s, 3 H, CH_3_); IR (CHCl_3_) 3427, 1658 cm^−1^; MS (EIS) m/z 463.26 (MH^+^). Anal. Calcd for C_28_H_26_N_6_O: C, 72.71; H, 5.67; N, 18.17. Found: C, 72.86; H, 5.46; N, 18.23.

##### 2.1.6.8. 3-(2-(5-(6-*n*-Butylpyridin-2-yl)-4-(quinoxalin-6-yl)-1*H*-imidazol-2-yl)ethyl)benzamide (16h)

Yield 18%; mp 115−118 °C; ^1^H NMR (CDCl_3_) δ 8.79 (s, 2 H, 2 quinoxalinyl), 8.32 (t, 1 H, quinoxalinyl), 8.05 (m, 2 H, 2 quinoxalinyl), 7.56 (m, 1 H, phenyl), 7.52 (s, 1 H, phenyl), 7.47 (t, 1 H, pyridyl), 7.34 (d, 1 H, pyridyl), 7.23 (overlapped, 1 H, phenyl), 7.19 (t, 1 H, phenyl), 7.00 (dd, 1 H, pyridyl), 6.90 (br s, 1 H, CONH), 6.23 (br s, 1 H, CONH), 3.04 (m, 2 H, CH_2_), 2.94 (m, 2 H, CH_2_), 2.66 (t, 2 H, CH_2_), 1.48 (m, 2 H, CH_2_), 1.23 (m, 2 H, CH_2_), 0.78 (t, 3 H, CH_3_); IR (CHCl_3_) 1657 cm^−1^; MS (EIS) *m/z* 477.30 (MH^+^). Anal. Calcd for C_29_H_28_N_6_O: C, 73.09; H, 5.92; N, 17.63. Found: C, 72.88; H, 6.15; N, 17.55.

### 2.2. Luciferase reporter assay

To establish HaCaT (3TP-luc) stable cells, cells were seeded on six-well plates. Cells were allowed to adhere overnight and then transfected with the p3TP-luc (neo) expression plasmid using PEI reagent (Sigma Aldrich). Transfected cells were cultured for four weeks in the presence of G418 (500 µg/mL). Several single clones were isolated and measured luciferase activity. The clone showing response to TGF-β1 treatment was used for reporter assay. HaCaT (3TP-luc) stable cells were seeded at 2.5 × 10^4^ cells/well in 96-well plate and were allowed to adhere overnight. Cells were concomitantly treated with TGF-β1 (2 ng/mL) and indicated concentrations of ALK5 inhibitors in 0.2% FBS medium and incubated for 24 h at 37 °C in 5% CO_2_. Cell lysates were prepared using Luciferase Assay System (Promega) according to the manufacturer’s instruction, and luminescence was measured by a luminometer, Micro Lumat Plus (Berthold, Germany).

### 2.3. Cell permeability assay

Caco-2 cells were seeded in Transwell^®^ polycarbonate filter at a density of 8 × 10^4^ cells/filter and cultured for 21 days. Culture medium was removed from both apical (AP) and basolateral (BL) chambers of transwell, and the wells were rinsed three times with PBS. AP buffer (HBSS, pH 6.5) containing 10 mM MES and BL buffer (HBSS, pH 7.4) containing 10 mM HEPES were loaded in AP (500 µL) and BL (1500 µL) chambers, respectively, followed by incubation for 30 min at 37 °C. Then, test compound (100 µM) was added to the AP side and incubated for 2 h at 37 °C. After the incubation, BL buffer was collected and analysed using an UV spectrophotometer at a maximum wavelength (wavelength 225–357 nm).

### 2.4. Docking study

All computational works were performed on the Sybyl-X 2.1.1 (Tripos Inc., St Louis, MO, USA) molecular modelling package with CentOS Linux 5.4. operating system1^27^.

#### 2.4.1. Preparation of ligands and receptor

The **11e** was prepared with sketch module embedded in Sybyl package and saved as mol2 format. After sketching the molecule, Gasteiger-Hückel charges were assigned to all atoms. To optimise the ligand, energy minimisation was conducted by the standard tripos force field with convergence to maximum derivatives of 0.001 kcal mol^−1^·Å^−1^. The X-ray structure of ALK5 complexed with 5,6-dihydro-4*H*-pyrrolo[1,2-*b*]pyrazole inhibitor was used as a receptor for docking (PDB id: 1RW8)^28^. Receptor structure was retrieved from PDB (http://www.rcsb.org/) and optimised using structure preparation tool embedded in biopolymer module. Native ligand was extracted and all water molecules except key water molecule for water-mediated hydrogen bond network were deleted from the complex structure.

#### 2.4.2. Molecular docking

To examine the binding poses of **11e**, docking study was conducted using Surflex-Dock3 embedded in Tripos Sybyl X 2.1.1 software package. For docking performance, the active site was assigned as a protomol generated by using the native ligand in the X-ray structure. Flexible docking was carried out by using default parameter values (threshold = 0.5 and bloat = 0), producing 200 conformers as maximum number of poses per ligand. Binding affinity of each docking pose of ligand was calculated by Surflex-dock score and consensus scoring function (CScore). The total Surflex-Dock score was expressed as –logKd to represent binding affinities. To build the best docking model, key interactions between candidate compound and active site were investigated by visual inspection.

## 3. Results and discussion

### 3.1. Chemistry

To develop a potent, selective, and orally bioavailable ALK5 inhibitor, as our starting point, we designed three target molecules, 4-((4-(benzo[1,3]dioxol-5-yl)-5-(6-methylpyridin-2-yl)-1*H*-imidazol-2-yl)methyl)benzamide (**7a**) and its ethyl and *n*-propyl analogues, **7b** and **7c** to optimise the distance between the two pharmacophores, a central imidazole ring and a phenyl ring.

**Scheme 1.**
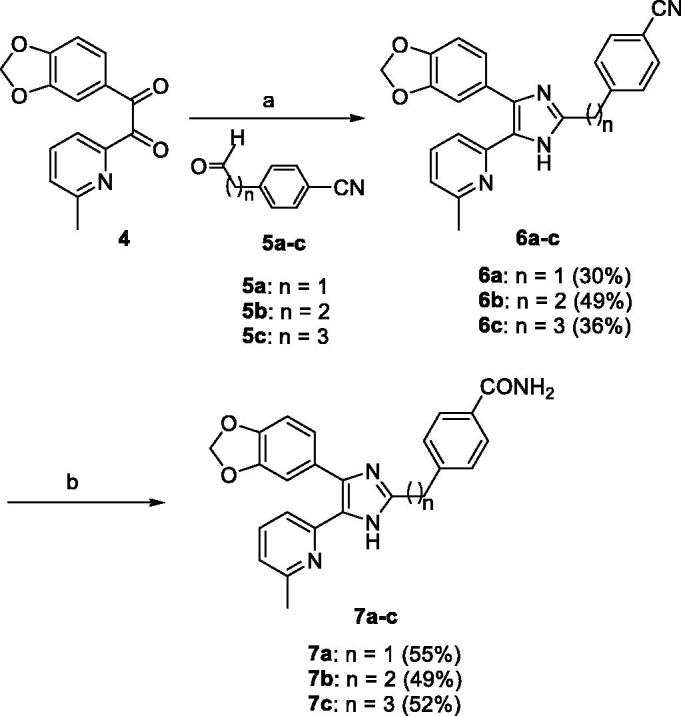
Reagents and conditions: (a) NH_4_OAc, AcOH, 120 °C, 3 h; (b) 28% H_2_O_2_, 6 N NaOH, EtOH, 55 °C, 3 h.

The **7a–c** were prepared as shown in Scheme 1. The 1-(benzo[1,3]dioxol-5-yl)-2-(6-methylpyridin-2-yl)ethane-1,2-dione (**4**)^29^ was condensed with 4-(2-oxoethyl)benzonitrile (**5a**)^30^, 4-(3-oxopropyl)benzonitrile (**5b**)^31^, or 4-(4-oxobutyl)benzonitrile (**5c**)^32^ and NH_4_OAc in AcOH at 120 °C to produce the 2-((4-cyanophenyl)alkyl)imidazoles **6a–c** in 30–49% yields. Transformation of the carbonitrile group of **6a–c** to the corresponding carboxamide group was accomplished by treatment with 28% H_2_O_2_ and 6 N NaOH in EtOH at 55 °C to provide the **7a–c** in 49–55% yields.

Since a methylene or an ethylene linker was proved to be more beneficial than a *n*-propylene linker in ALK5 inhibition in both a kinase assay and a cell-based luciferase reporter assay, we next synthesised a series of 5-(6-alkylpyridin-2-yl)-4-(quinoxalin-6-yl)-1*H*-imidazoles, **11a–h** and **16a–h**, to compare the impact of a benzo[1,3]dioxol-5-yl moiety and a quinoxalin-6-yl moiety in ALK5 inhibition.

**Scheme 2.**
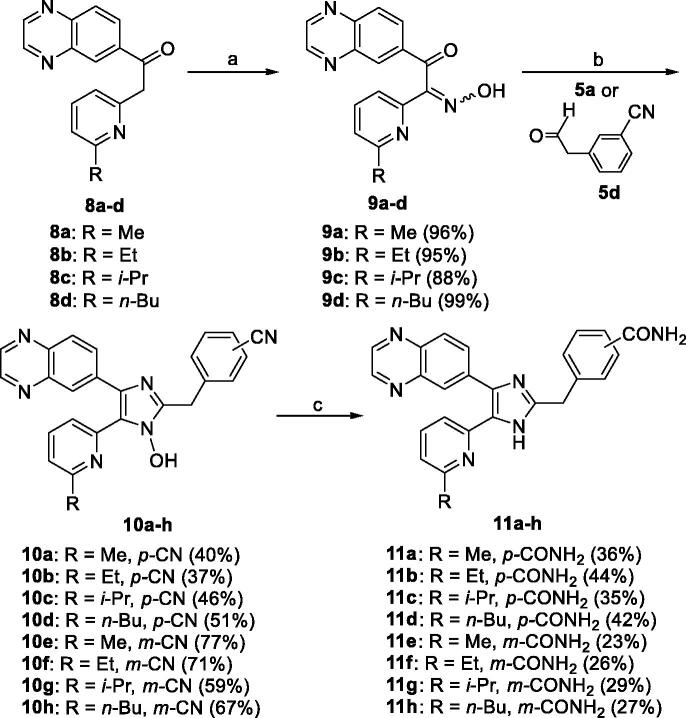
Reagents and conditions: (a) NaNO_2_, 5 N HCl, rt, 1 h; (b) **5a** or 3-(2-oxoethyl)benzonitrile (**5d**), NH_4_OAc, *t*-BuOMe/MeOH, rt, overnight, Ar atmosphere; (c) (i) 28% H_2_O_2_, 6 N NaOH, EtOH, DMSO, 55 °C, overnight; (ii) triethyl phosphite, anhydrous DMF, 110 °C, 72 h.

The **11a–h** were prepared as shown in Scheme 2. The 2-(6-alkylpyridin-2-yl)-1-(quinoxalin-6-yl)ethanones **8a–d**^33^ were treated with NaNO_2_ in 5 N HCl to give the 2-(6-alkylpyridin-2-yl)-2-(hydroxyimino)-1-(quinoxalin-6-yl)ethanones **9a–d** in 88–99% yields. Condensation of **9a–d** with either **5a** or 3-(2-oxoethyl)benzonitrile (**5d**)^34^ and NH_4_OAc in a mixture of *t*-BuOMe and MeOH at room temperature afforded the 5-(6-alkylpyridin-2-yl)-1-hydroxy-4-(quinoxalin-6-yl)-1*H*-imidazoles **10a–h** in 37–77% yields. Conversion of the carbonitrile group of **10a–h** to the carboxamide group and subsequent dehydroxylation with triethyl phosphite in anhydrous DMF at 110 °C for 3 days gave the target compounds **11a–h** in 23–44% yields.

**Scheme 3.**
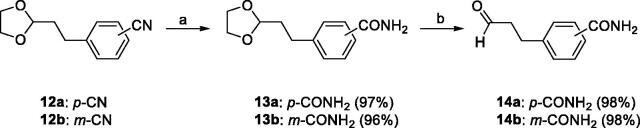
Reagents and conditions: (a) 28% H_2_O_2_, 6 N NaOH, MeOH, 55 °C, 2 h; (b) 1 N HCl, THF, reflux, 1 h.

The requisite aldehydes, 4–(3-oxopropyl)benzamide (**14a**) and 3-(3-oxopropyl)benzamide (**14b**) for the synthesis of the target compounds **16a–h** were prepared as shown in Scheme 3.

**Scheme 4.**
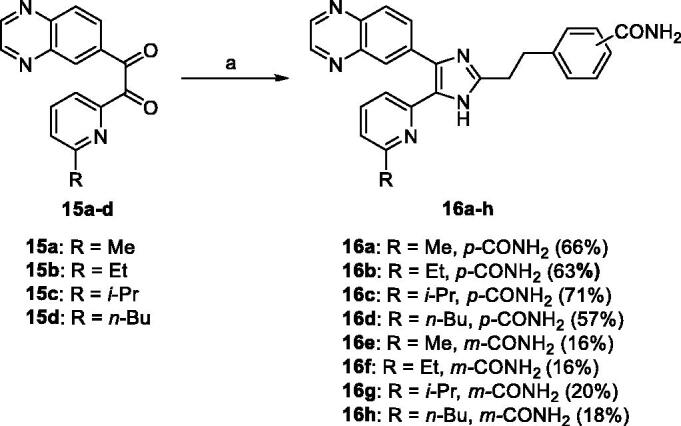
Reagents and conditions: (a) **14a** or **14 b**, NH_4_OAc, *t*-BuOMe/MeOH, 30 °C, overnight, Ar atmosphere.

Selective hydrolysis of the carbonitrile group of **12a**^31^ and **12b**^35^ to the carboxamide group followed by acidic hydrolysis of 1,3-dioxolanyl protecting group of **13a** and **13 b** afforded **14a** and **14 b** in almost quantitative yield. The 2-(6-alkylpyridin-2-yl)-1-(quinoxalin-6-yl)ethane-1,2-diones **15a–d**^33^ was condensed with either **14a** or **14 b** and NH_4_OAc in a mixture of *t*-BuOMe and MeOH to obtain the 4-carboxamide analogues **16a–d** in modest yields (57–71%) and the 3-carboxamide analogues **16e–h** in lower yields (16–20%) (Scheme 4).

### 3.2. ALK5 inhibitory activity in an enzyme assay and in a cell-based luciferase reporter assay

To evaluate whether these potential inhibitors **7a–c**, **11a–h**, and **16a–h** could inhibit ALK5, a kinase assay was performed using the purified human ALK5 kinase domain produced in Sf9 insect cells and casein as a substrate (Table 1). The ALK5 inhibitory activity of **7b** (IC_50_ = 0.093 µM) was 2.4-fold and 3.6-fold higher than those of **7a** (IC_50_ = 0.224 µM) and **7c** (IC_50_ = 0.338 µM), respectively, in a kinase assay (Table 1). In a cell-based luciferase reporter assay using HaCaT (3TP-luc) stable cells, **7c** (40% inhibition) was much less inhibitory than **7a** (72% inhibition) and **7b** (74% inhibition) at a concentration of 0.1 µM.

**Table 1.** ALK5 inhibitory activity and Caco-2 cell permeability of 2,4-disubstitubed-5–(6-alkylpyridin-2-yl)-1*H*-imidazoles **7a–c**, **11a–h**, and **16a–h**.
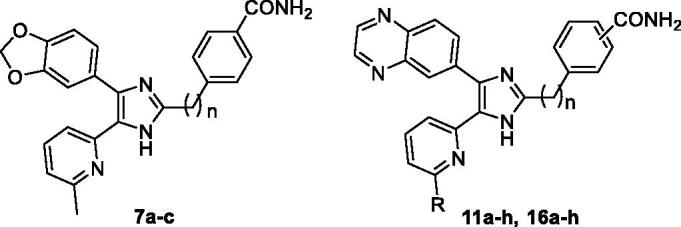

Compound	*R*	CONH_2_	*n*	IC_50_^a,b^ (μM)	p3TP-luciferase activity^d^ (% control)	Caco-2 permeability (%)
Mock					2 ± 0^e^	
TGF-β					100 ± 5.0	
**7a**			1	0.224	28 ± 7.1	35 ± 0.7^f^
**7b**			2	0.093	26 ± 7.9	23 ± 3.0
**7c**			3	0.338	60 ± 9.5	32 ± 1.1
**11a**	Me	*p*-CONH_2_	1	0.088	16 ± 4.5	25 ± 1.3
**11b**	Et	*p*-CONH_2_	1	0.112	33 ± 21.3	26 ± 3.5
**11c**	*i*-Pr	*p-*CONH_2_	1	2.195	39 ± 11.4	30 ± 9.0
**11d**	*n*-Bu	*p-*CONH_2_	1	2.700	85 ± 32.5	41 ± 5.3
**11e**	Me	*m-*CONH_2_	1	0.036^c^	8 ± 5.8	56 ± 26.8
**11f**	Et	*m-*CONH_2_	1	0.075	17 ± 3.7	38 ± 13.8
**11g**	*i*-Pr	*m-*CONH_2_	1	1.250	69 ± 6.7	34 ± 15.6
**11h**	*n*-Bu	*m-*CONH_2_	1	1.038	83 ± 21.1	n.d.^g^
**16a**	Me	*p-*CONH_2_	2	0.030	15 ± 8.2	45 ± 5.3
**16b**	Et	*p-*CONH_2_	2	0.041	16 ± 8.4	25 ± 3.7
**16c**	*i*-Pr	*p-*CONH_2_	2	0.653	85 ± 38.9	23 ± 3.1
**16d**	*n*-Bu	*p-*CONH_2_	2	2.383	76 ± 5.7	24 ± 1.9
**16e**	Me	*m-*CONH_2_	2	0.047	7 ± 0.7	21 ± 9.5
**16f**	Et	*m-*CONH_2_	2	0.066	56 ± 14.6	6 ± 4.1
**16g**	*i*-Pr	*m-*CONH_2_	2	0.593	48 ± 41.8	0
**16h**	*n*-Bu	*m-*CONH_2_	2	2.875	92 ± 10.4	0

^a^ALK5 was expressed in Sf9 insect cells as human recombinant GST-fusion protein by means of the vaculovirus expression system. A Proprietary radioisotopic protein kinase assay (^33^PanQinase^®^ Activity Assay) was performed at ProQinase GmbH (Freiburg, Germany). The assay contained 60 mM HEPES-NaOH, pH 7.5, 3 mM MgCl_2_, 3 mM MnCl_2_, 3 μM Na-orthovanadate, 1.2 mM DTT, 50 μg/mL PEG_20000_, 1 μM [γ-^33^P]-ATP (approximately 5 × 10^5^ cpm per well). One hundred ng/50 μL of ALK5 and 1000 ng/50 μL of casein as a substrate were used per well.

^b^Singlicate.

^c^Mean of triplicates.

^d^HaCaT (p3TP-luc) stable cells were used. Luciferase activity was determined at a concentration of 0.1 μM of inhibitor.

^e^The mean ± SD of three independent experiments run in triplicates relative to control incubations with DMSO vehicle.

^f^The mean ± SD of quadruplicates.

^g^Not determined.

Replacement of a benzo[1,3]dioxol-5-yl moiety in **7a** and **7b** with a quinoxalin-6-yl moiety increased ALK5 inhibitory activity, thus, the corresponding analogues, **11a** (IC_50_ = 0.088 µM) and **16a** (IC_50_ = 0.030 µM), were 2.5-fold and 3.1-fold more potent than **7a** and **7b**, respectively, in a kinase assay (Table 1). Similar to kinase assay, **11a** (84% inhibition) and **16a** (85% inhibition) were much more inhibiting than **7a** and **7b** in a luciferase reporter assay. It was previously demonstrated that a Me substituent at the six-position of the pyridine ring in the pyridyl-substituted ALK5 inhibitors significantly increased ALK5 inhibitory activity^36–38^. Therefore, we examined the effect of bulkier alkyl groups such as Et, *i*-Pr, and *n*-Bu in ALK5 inhibition. The 6-ethylpyridyl analogues **11b**, **11f**, **16b**, and **16f** were 1.3-, 2.1-, 1.4-, and 1.4-fold less potent than the corresponding 6-methylpyridyl analogues **11a**, **11e**, **16a**, and **16e**, respectively. However, the 6-*i*-propylpyridyl analogues (**11c**, **11g**, **16c**, and **16g**) and 6-*n*-butylpyridyl analogues (**11d**, **11h**, **16d**, and **16h**) displayed 13–35-fold and 29–71-fold lower inhibitory activity compared to the respective 6-methylpyridyl analogues, indicating that a bulkier group than Et group cannot be accommodated favourably into the ATP binding pocket of ALK5. Regarding the length of a linker, an ethylene linker was generally more beneficial than a methylene linker in the ALK5 inhibition as shown in **7a** and **7b**, thus, **16a**, **16b**, **16c**, and **16g** were 2.9-, 2.7-, 3.4-, and 2.1-fold more inhibiting than the respective **11a**, **11b**, **11c**, and **11g**. Compounds **16d–f** showed the similar level of potency to that of **11d–f**, respectively. The position of a carboxamide group in the phenyl ring also influenced ALK5 inhibition. The *m*-CONH_2_ analogues **11e–h** were 1.5–2.6-fold more inhibiting than the respective *p*-CONH_2_ analogues **11a–d** in a series of compounds having a methylene linker, whereas the *p*-CONH_2_ analogues having an ethylene linker, **16a** and **16 b**, were 1.6-fold more inhibiting than the corresponding *m*-CONH_2_ analogues **16e** and **16f**. Compounds showing IC_50_ values of <0.05 µM in a kinase assay, **11e** (IC_50_ = 0.036 µM), **16a**, **16 b** (IC_50_ = 0.041 µM), and **16e** (IC_50_ = 0.047 µM) exhibited much higher inhibitory activity in a luciferase reporter assay, also (**11e**: 92%, **16a**: 85%, **16b**: 84%, **16e**: 93%).

Among this series of compounds, **11e** possessing the most potent ALK5 inhibitory activity and the highest permeability in Caco-2 cells was selected, and its ALK5 inhibitory activity was compared with those of potential competitors, galunisertib and vactosertib in a kinase assay and in a luciferase reporter assay. In a kinase assay, **11e** (IC_50_ = 0.013 µM) showed the same level of potency to that of vactosertib (IC_50_ = 0.013 µM) and 6.6-fold higher potency compared to that of galunisertib (IC_50_ = 0.086 µM) (Table 2). Luciferase activity of HaCaT (3TP-luc) cells was increased by 65-fold after treatment of TGF-β1 (2 ng/mL), and **11e** and vactosertib displayed the similar level of inhibition on the TGF-β1-induced luciferase reporter activity with IC_50_ values of 0.0196 µM and 0.0165 µM, respectively. Similar to a kinase assay, galunisertib displayed much lower inhibition (IC_50_ >0.1 µM) compared to **11e** and vactosertib (Table 2).

**Table 2.** ALK5 and p38α inhibitory activity of **11e**, **1**, and **2**.

Compound	IC_50_ for ALK5 (μM)^a,b^	IC_50_ for p38α (μM)^b,c^	Selectivity index^d^	IC_50_ for p3TP-luciferase (μM)^e,f^
**11e**	0.013	0.288	22	0.0196
Galunisertib **(1)**	0.086	0.320	4	>0.1
Vactosertib **(2)**	0.013	1.775	137	0.0165

^a^A proprietary radioisotopic protein kinase assay (^33^PanQinase^®^ Activity Assay) was performed at ProQinase GmbH (Freiburg, Germany). The assay contained 70 mM HEPES-NaOH, pH 7.5, 3 mM MgCl_2_, 3 mM MnCl_2_, 3 μM Na-orthovanadate, 1.2 mM DTT, 50 μg/mL PEG_20000_, 1 μM [γ-^33^P]-ATP (approximately 6 × 10^5^ cpm per well). Five ng/50 μL of ALK5 and 1000 ng/50 μL of GSK3 (14–27) as a substrate were used per well.

^b^Mean of duplicates.

^c^A proprietary radioisotopic protein kinase assay (^33^PanQinase^®^ Activity Assay) was performed at ProQinase GmbH (Freiburg, Germany) using ATF2 as a substrate.

^d^IC_50_ for p38α/IC_50_ for ALK5.

^e^HaCaT (3TP-luc) stable cells were used.

^f^Mean of triplicates.

The kinase domain of p38α is known to be one of the most homologous to that of ALK5^39^, therefore, this enzyme was chosen to compare selectivity of **11e**, galunisertib, and vactosertib. In a p38α kinase assay, IC_50_ values of **11e**, galunisertib, and vactosertib were 0.288 µM, 0.320 µM, and 1.775 µM, respectively, thus, their selectivity indices (IC_50_ for p38α/IC_50_ for ALK5) were 22, 4, and 137, respectively. Although **11e** was 5.5-fold more selective against p38α than galunisertib, it was 6.2-fold less selective than vactosertib.

### 3.3. Caco-2 cell permeability assay

To estimate oral absorption of target compounds, their permeability in a Caco-2 monolayer was evaluated at a concentration of 100 µM (Table 1). The **11e** showed the highest permeability (56%), but **16f** (6%), **16g** (0%), and **16h** (0%) showed very limited or no permeation in this assay, demonstrating that even simple structural modification markedly affected the permeability in this series of compounds.

### 3.4. Kinase profiling assay

**Figure 2.**
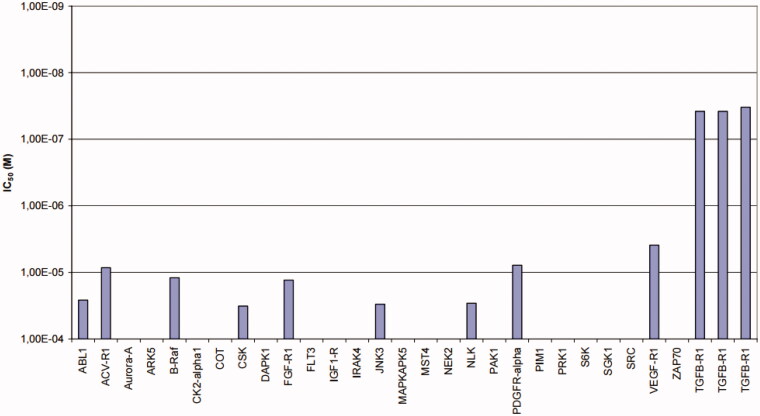
Inhibitory profile of **11e** in 28 protein kinase assays.

Considering the ALK5 inhibitory activity in both a kinase assay and a luciferase reporter assay and the Caco-2 cell permeability, **11e** was selected as a candidate for preliminary kinase profiling. Because ALK5 is a serine/threonine kinase, we chose 19 serine/threonine kinases including ALK5 and an ALK family kinase, ACV-R1 (ALK5, ACV-R1, Aurora-A, ARK5, B-Raf, CK2α1, COT, DAPK1, IRAK4, JNK3, MAPKAPK5, MST4, NEK2, NLK, PAK1, PIM1, PRK1, S6K, SGK1) and 9 tyrosine kinases (ABL1, CSK, FGF-R1, FLT3, IGF1-R, PDGFR-α, SRC, VEGF-R1, ZAP70). Selectivity profiling of **11e** using a panel of 28 protein kinases showed selectivity indices of >100 against all the kinases tested (ProQinase GmbH (Freiburg, Germany)) (Figure 2).

### 3.5. Docking model of ALK5 in complex with 11e

**Figure 3.**
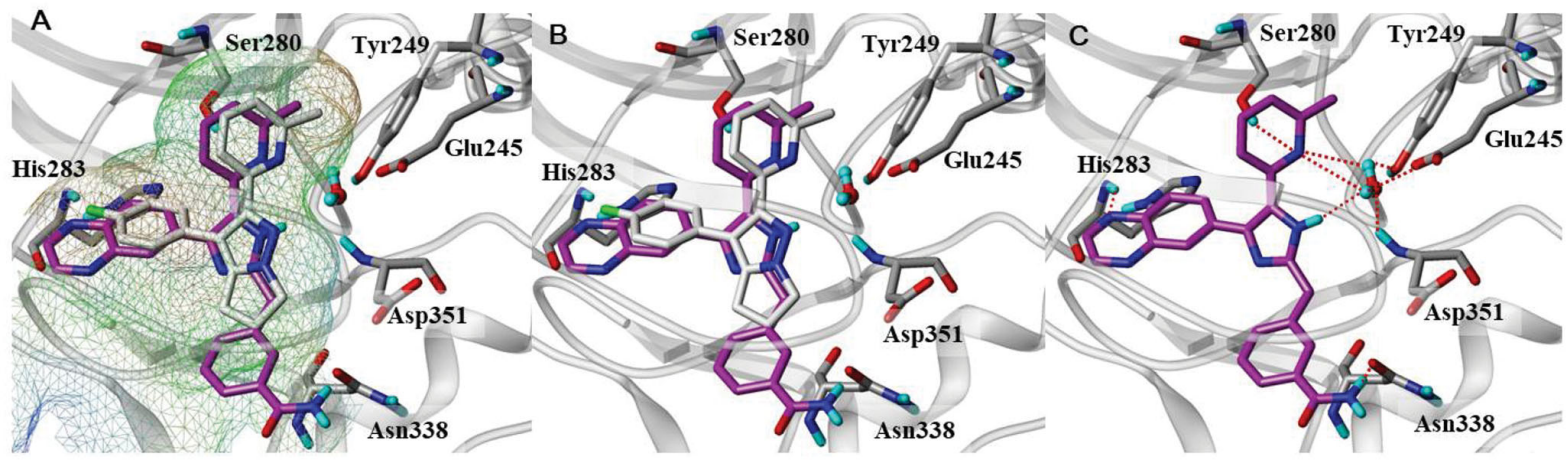
Docked pose of **11e** in the active site of ALK5 (PDBid:1RW8). (A, B) **11e** is (magenta carbon atoms) superimposed over the X-ray pose of native ligand (grey carbon atoms). The active site of ALK5 is shown as MOLCAD lipophilic potential surface map (A), and lipophilicity increases from blue (hydrophilic) to brown (liphophilic). Grey capped sticks represent key amino acid residues within the binding site, and the backbone of ALK5 is shown as ribbon. The bound water molecule in the X-ray structure is represented by ball and stick. (C) Intermolecular interaction between **11e** and ALK5. Grey capped sticks represent key amino acid residues in the active site. Red dashed lines are hydrogen bonding interactions (<3.0 Å).

To determine the binding pose of **11e** in the active site of ALK5, we executed docking modelling with flexible molecular docking programme Surflex-dock^40^. We analysed the result of docking considering Surflex-dock docking score (–logKd) and consensus score (obtained from CScore module^27^), and selected the poses of **11e** with high scores (–logKd ≥7 and CScore ≥3). To select the best docked pose among them, we also identified key interactions between amino acid residues in the active site and **11e**, in comparison with the X-ray pose of native ligand (3-(4-fluorophenyl)-2-(6-methylpyridin-2-yl)-5,6-dihydro-4*H*-pyrrolo[1,2-*b*]pyrazole)^28^. As shown in Figure 3(A,B), **11e** fits well into cavity of active site, with quinoxaline ring and methyl group of 6-methylpyridine ring occupying the hydrophobic pockets. Hydrogen bond interactions between **11e** and ALK5 are exhibited in Figure 3(C). The quinoxaline ring nitrogen acts as a hydrogen bond acceptor and interacts with NH of His283 in the backbone of hinge region of ALK5. The 6-methylpyridine ring nitrogen forms a water-mediated hydrogen bond network with the backbone NH of Asp351, and the side chain of both Tyr249 and Glu245. The imidazole ring NH of **11e** is also involved in the water-mediated hydrogen bond network. The NH_2_ group of carboxamide in the *meta* position of phenyl ring interacts with the side chain of Asn338 by forming hydrogen bond, which cannot be formed for the compounds, **11a–d** having the carboxamide group in the *para* position. Our docking model for the most active compound **11e** well supports the key interactions for ALK5 inhibition which was previously reported^28^.

## 4. Conclusions

In this report, a series of 2,4-disubstituted-5-(6-alkylpyridin-2-yl)-1*H*-imidazoles, **7a–c**, **11a–h**, and **16a–h** has been synthesised and evaluated for their ALK5 inhibitory activity in an enzyme assay and in a cell-based luciferase reporter assay. The structure**–**activity relationships in this series of compounds revealed that an ethylene linker at the two-position of the imidazole ring was the most beneficial in ALK5 inhibitory activity. Replacement of a benzo[1,3]dioxol-5-yl moiety at the four-position of the imidazole ring with a quinoxalin-6-yl moiety markedly increased ALK5 inhibitory activity. Regarding the alkyl substituent at the six-position of the pyridine ring, the compounds having a methyl or an ethyl substituent displayed much higher inhibitory activity than the compounds having an *i*-propyl or a *n*-butyl substituent. The *m*-CONH_2_ analogues were more inhibiting than the *p*-CONH_2_ analogues in compounds having a methylene linker, whereas the *p*-CONH_2_ analogues were more inhibiting than the *m*-CONH_2_ analogues in compounds having an ethylene linker. In a cell permeability assay using Caco-2 monolayer, **11e** showed the highest permeability in this series of compounds. The **11e** was equipotent to vactosertib, but much more potent than galunisertib in an ALK5 kinase assay and in a cell-based luciferase reporter assay. Although **11e** was 5.5-fold more selective against p38α than galunisertib, it was 6.2-fold less selective than vactosertib. Therefore, it can be concluded that combination of replacement of a [1,2,4]triazolo[1,5-*a*]pyridin-6-yl moiety with a quinoxalin-6-yl moiety, insertion of a methylene linker instead of a methyleneamino linker, and a *m*-CONH_2_ substituent in the phenyl ring in vactosertib maintained its high ALK5 inhibitory activity, but decreased its selectivity against p38α. Selectivity profiling of **11e** using a panel of 28 protein kinases showed that it is highly selective for ALK5. Our docking results demonstrate that **11e** fits well in the ATP-binding pocket of ALK5 with favourable intermolecular interactions.
